# Practical Considerations for the Use of the Rapid AcuStar^®^ ADAMTS13 Activity Assay in the Diagnosis of Acute Thrombotic Thrombocytopenic Purpura (TTP)

**DOI:** 10.3390/jcm13154462

**Published:** 2024-07-30

**Authors:** Jun Yong, Stephen MacDonald, Colin Downey, Rebekah Fretwell, Caroline Lawrence, Paul Murphy, Thomas Pitchford, Tina Dutt

**Affiliations:** 1 Department of Clinical Infection, Microbiology and Immunology, University of Liverpool, Liverpool L69 3BX, UK; j.x.yong@liverpool.ac.uk; 2 The Roald Dahl Centre for Haemostasis and Thrombosis, Liverpool University Hospital NHS Trust, Liverpool L7 8XP, UK; colin.downey@liverpoolft.nhs.uk; 3 Cambridge Haemophilia and Thrombophilia Centre, Cambridge University Hospitals NHS Foundation Trust, Cambridge CB2 0QQ, UK; stephenmacdonald1@nhs.net; 4 Department of Coagulation, Sheffield Teaching Hospitals NHS Foundation Trust, Sheffield S10 2JF, UK; r.fretwell@nhs.net (R.F.); thomas.pitchford@nhs.net (T.P.); 5 Department of Haemostasis, Glasgow Royal Infirmary, Glasgow G4 0SF, UK; caroline.lawrence@ggc.scot.nhs.uk; 6 Haematology and Haemostasis, The Newcastle Upon Tyne Hospitals NHS Foundation Trust, Newcastle upon Tyne NE3 3HD, UK

**Keywords:** thrombotic thrombocytopenic purpura, ADAMTS13, ELISA, enzyme assay, diagnosis

## Abstract

**Introduction:** Conventional practice in the management of acute TTP entails empirical treatment of suspected cases whilst awaiting confirmatory ADAMTS13 deficiency testing. Rapid ADAMTS13 assays offer increased accessibility and rapid diagnostics. The new automated HemosIL AcuStar^®^ ADAMTS13 assay has seen increasing use among UK TTP Specialist Centres alongside the traditional ELISA method to confirm severe ADAMTS13 deficiency. **Methods:** A multi-centre retrospective case-control study was performed to review patients demonstrating discrepant ADAMTS13 activity results measured using rapid (AcuStar^®^) and ELISA assays in parallel from September 2019 to December 2021. Cases were compared with a cohort of suspected TTP patients exhibiting no difference in assay results and in relation to their presenting characteristics and pre-test probability of a diagnosis of TTP. **Results:** Where the clinical index of suspicion for TTP was high at presentation, acute TTP was confirmed using the AcuStar^®^ assay < 0.2 IU/dL and subsequently < 10 IU/dL by ELISA with zero incidence of discrepancy. For patients with low clinical suspicion of acute TTP, a discrepancy between the AcuStar^®^ and ELISA assay results was observed in 2% of cases; 5–10 IU/dL in AcuStar^®^, confirmed as >20 IU/dL by ELISA. A concurrent cancer diagnosis or sepsis was observed in 40% of discrepant cases. **Conclusions:** Where acute TTP is strongly suspected, there is a good correlation between the rapid AcuStar^®^ ADAMTS13 assay and the conventional ELISA assay. Where the clinical suspicion of acute TTP is low, caution should be exercised in the interpretation of the ADAMTS13 activity using the AcuStar^®^ assay. Accurate interpretation requires robust ADAMTS13 testing algorithms to be incorporated into diagnostic pathways.

## 1. Introduction 

A severe deficiency of ADAMTS13 (a disintegrin and metalloproteinase with a thrombospondin type 1 motif, member 13) activity < 10 IU/dL is diagnostic of thrombotic thrombocytopenia purpura (TTP) [1,2] and occurs as a result of a congenital deficiency or antibody-mediated autoimmune destruction of ADAMTS13 [1]. 

TTP is a haematological emergency with high mortality if untreated [3]. This time-dependent lethality is reduced significantly by prompt diagnosis and treatment with plasma exchange (PEX) [4,5]. PEX restores circulating ADAMTS13 levels while removing accumulated ultra-large von Willebrand factor (vWF) multimers and, in the case of immune-mediated TTP (iTTP), ADAMTS13 autoantibodies. Following diagnostic confirmation, caplacizumab, a disease-modifying therapy, may be administered acutely to provide a window of time for the effects of immunotherapy, usually the anti-CD20 monoclonal antibody rituximab, to induce a remission of iTTP [4,6].

Prompt, outcome-influencing decision-making is dependent on a high index of clinical suspicion for a diagnosis of TTP, combined with a reliable, confirmatory diagnostic test [1,7]. Evidence-based predictive scoring systems such as the French thrombotic microangiopathy (TMA) score and the prospectively validated PLASMIC score are variably employed by clinicians in this process [8,9,10]. Pending verification of a diagnosis of acute TTP, conventional wisdom advises empirical initiation of PEX [2,3]. Confirmation of a severe ADAMTS13 deficiency is highly sensitive and specific for a TTP diagnosis [2,11] and accelerates specialist TTP management, avoiding blind PEX or adjuvant therapies where the diagnosis is excluded [12].

Conventional laboratory methods of ADAMTS13 activity quantification are paradoxically time-consuming and labour-intensive [2,11]. ADAMTS13 assays typically demonstrate the innate capability of plasma to cleave full-length or truncated vWF fragments containing the ADAMTS13 cleavage site ex vivo [11]. Fluorescence and chromogenic-based assays are available commercially [fluorescence resonance energy transfer (FRET) and enzyme-linked immunosorbent assay (ELISA), respectively], although expense and high technical demand restrict employment to specialised laboratories [11]. Currently, most United Kingdom (UK) laboratories measure ADAMTS13 activity by ELISA. However, rapid ADAMTS13 quantification methods have more recently emerged, including assays by Technoclone, Austria (Technoscreen and Technoflour) and Werfen (Bedford, NY, USA) [13,14,15]. 

Werfen’s chemiluminescent-based rapid assay, developed on their AcuStar^®^ platform, utilises an isoluminol-labelled monoclonal antibody that interacts with cleaved vWF73 isolated on magnetic particles to elicit a chemiluminescent signal proportional to the amount of ADAMTS13 activity, promising an ADAMTS13 activity result within 30 min [15,16]. The assay is highly sensitive for ADAMTS13 levels < 10 IU/dL and has comparable positive predictive values for TTP when compared to conventional methods [15]. 

Increased use of the AcuStar^®^ assay as a screening test for suspected acute TTP has revealed a discrepancy between results obtained using the AcuStar^®^ versus the conventional ELISA assay [17,18]. This communication reports the shared experience of several United Kingdom (UK) TTP Regional Treatment Centres performing the standard ELISA assay alongside the rapid AcuStar^®^ ADAMTS13 assay for cases of suspected acute TTP. Discrepant ADAMTS13 activity results present a clinical conundrum for teams endeavouring to establish an urgent diagnosis of TTP with significant therapeutic implications. The important practical considerations for laboratories and clinical teams involved in the accurate diagnosis are discussed here.

## 2. Methodology

This was a retrospective multi-centre study performed by 5 UK TTP treating centres adopting rapid ADAMTS13 testing on the AcuStar^®^ platform in conjunction with the ADAMTS13 ELISA assay for referred cases of suspected acute TTP diagnosis between September 2019 and December 2021. 

Discrepant results were identified based on clinically unexpected low ADAMTS13 activity results by HemosIL AcuStar^®^ compared to Technozym^®^ ELISA. A discrepancy was defined as results < 10 IU/dL by rapid method compared to >10 IU/dL by Technozym^®^ ELISA. Samples were tested either as part of real-time sequential parallel testing for the purposes of method verification or in real-time post implementation of the rapid assay in centres. In the case of the latter, the use of the rapid method was followed by confirmation with Technozym^®^ ELISA within 24 h. 

‘High clinical suspicion of TTP’ patients were defined as those documented as ‘likely TTP’ with justification by the clinician receiving the referral (laboratory markers/scoring system) or patients known to have a previous TTP diagnosis. Patients known to have congenital TTP or known Atypical Haemolytic Uraemic Syndrome were excluded. A control group of patients with non-discrepant results was taken from a convenience sample of suspected TTP requests during the same period where the clinical index of suspicion of TTP was high. The underlying demographics for patients presenting for testing during this period did not provide the opportunity to age and gender match discrepant and non-discrepant groups. However, testing performed on the control group was conducted during the same time period as the discrepant group.

Pre-agreed clinical and laboratory data were extracted from electronic patient records, as presented in Table 1. Where the requested data was not present in the notes, this is described in the figure legends.

The Plasmic score is a seven-component score, with one point designated for each component per category defined as either elevated reticulocyte count (>2.5%), undetectable haptoglobin or indirect (unconjugated) bilirubin > 2.0 mg/dL (34 µmol/L) as indicators of haemolysis, active cancer (treatment within the previous 12 months), and history of solid organ or stem cell transplant each score one point if present. Each platelet count < 30 × 10^9^/L, MCV < 90 fL, INR < 1.5 and creatinine < 2.0 mg/dL (<177 µmol/L) also score a point if present. The PLASMIC score stratifies patients into low-risk (0–4), intermediate-risk (5), and high-risk (6–7) categories. The French TMA score scores one point for platelet count < 30 × 10^9^/L, serum creatinine ≤ 200 µmol/L, detectable anti-nuclear antibodies [ANA]. Severe ADAMTS13 deficiency, defined as <5% normal activity, is associated with a score of one in any category.

During data analysis, a PLASMIC score was retrospectively applied based on submitted values and was therefore not available for all patients. Likewise, a French TMA score was retrospectively applied (excluding anti-nuclear antibodies, as not routinely performed).

Data collection had approval from the Research and Development or Audit departments of each Trust. The study was conducted in accordance with the Declaration of Helsinki, and the protocol was approved as a service evaluation by Cambridge University Hospitals NHS Foundation Trust (Project identification code 6343). The overall project was approved as a service evaluation project.

### 2.1. Assays 

#### 2.1.1. Technozym^®^ ADAMTS13 ELISA

The Technozym^®^ ADAMTS13 ELISA method (Technoclone, Vienna, Austria) calibrates cleavage of the Glutathione-S-Transferase (GST)-vWF73 substrate to ADAMTS13 activity through a proportional colourimetric change. All assays were performed in duplicate, in a single assay run, with results interpolated by a calibration curve constructed each assay run from a series of six diluted calibrators provided in each kit, traceable to an international standard (12/252 first WHO international standard for ADAMTS13 function and antigen, NIBSC, Potters Bar, UK). All assays were performed at each of the local centres, and data were collated centrally for analysis.

#### 2.1.2. ADAMTS13 Activity by Chemiluminescence Immunoassay (CIA)

The AcuStar^®^ HemosIL Chemiluminescent immunoassay (CIA) from Werfen (Bedford, NY, USA) uses magnetic particles within a two-step immuno-assay. This procedure advances ELISA-based methodologies to allow automation. The magnetic particles act as the solid phase to isolate the activity of the enzyme that is then detected by Chemiluminescence. The magnetic particles are themselves coated in GST-vWF73. An anti-GST antibody localises ADAMTS13 activity within the patient sample to promote proportional cleavage of the substrate, detected using isoluminol added in the second step—specific to the cleaved substrate activating it.

### 2.2. Statistical Analysis

Data was collected, stored, and reviewed in Microsoft Excel 365 (Microsoft). Data was analysed utilising the Mann-Whitney U and Chi-square tests on SPSS (IBM) and the statistical programming environment R [R Core Team (2021)].

## 3. Results

Between September 2019 and December 2021, 20 patients referred with suspected acute TTP were found to demonstrate a discrepancy in ADAMTS13 activity results between the AcuStar^®^ and ELISA assay methods. The characteristics of this group of patients are compared with the characteristics of 20 patients with non-discrepant ADAMTS13 activity results referred during the same period. These patients had a high pre-test probability of acute TTP (likely/known previous TTP; Table 1).

Patients found to have discrepant ADAMTS13 activity results were more likely to have multiple comorbidities or concurrent active diagnoses of cancer or infection (Table 2). Discrepancies ranged from 17.7 to 75.9 IU/dL difference between methods. The degree of difference was not concentration-dependent (based on ELISA results). Rapid results as a group were 22% of the ELISA result (median, IQR—19.7–32.2).

**Figure 1:**
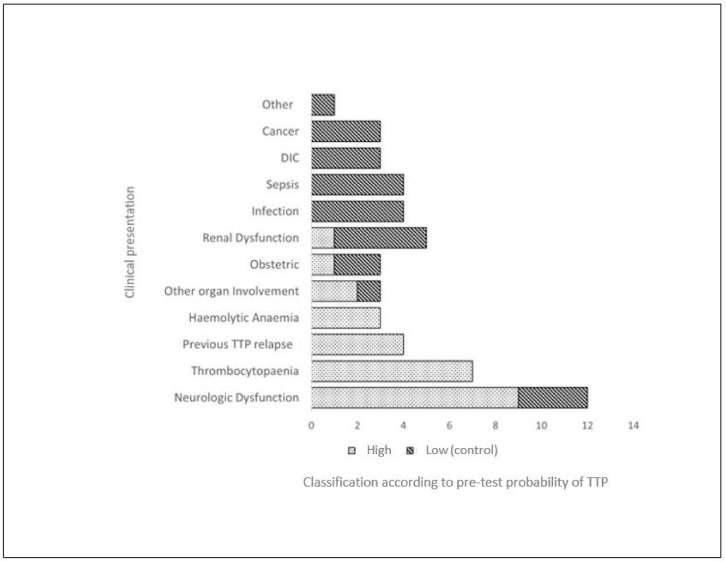
Analysis of clinical information accompanying patient samples forwarded for ADAMTS13 testing, discrepant ADAMTS13 (Dark shaded boxes) vs. control groups (Light shaded boxes). Retrospective cluster analysis of the clinical information distinguishing the control group with a high pre-test risk of TTP from the discrepant ADAMTS13 group with a low pre-test risk of TTP. ‘Other organ involvement’ excludes renal and neurological involvement. DIC, disseminated intravascular coagulation; TTP, thrombotic thrombocytopenic purpura.

A cluster analysis of the clinical information provided with patient samples referred for ADAMTS13 testing (Figure 1) highlights the differences in clinical presentation of patients that are associated with the pre-test probability of TTP. 

Patients with non-discrepant ADAMTS13 results had clinical features consistent with a high risk of TTP, irrespective of first-time presentation or at relapse. These were mainly neurological symptoms, thrombocytopaenia and haemolytic anaemia. In contrast, the presence of comorbidities, including cancer, disseminated intravascular coagulation (DIC) and infection in those investigated for microangiopathic haemolytic anaemia were more frequently found to demonstrate discrepant results. These are known to be associated with a low risk of TTP and were not seen in high-risk patients with non-discrepant ADAMTS13 results.

## 4. Discussion

### 4.1. Comparing ELISA and AcuStar^®^ ADAMTS13 Activity Results for the Diagnosis of Acute TTP

Rapid assays for ADAMTS13 quantification provide an opportunity for earlier diagnosis and prompt targeted therapy in TTP. The Werfen rapid AcuStar^®^ assay has demonstrated comparable sensitivity to conventional ADAMTS13 assays in pre-selected populations [15,19]. However, discrepancies between assays in a subpopulation of suspected TTP cases have only come to light with real-world use, particularly in their employment for negative prediction. 

The results of this report focus specifically on comparing the most widely used Technozym^®^ ADAMTS13 ELISA assay in UK TTP Centres with the new rapid AcuStar^®^ assay in the diagnosis of acute TTP. In patients where the clinical index of suspicion for TTP was high at presentation, acute iTTP was confirmed using the AcuStar^®^ assay < 0.2 IU/dL and subsequently < 10 IU/dL by Technozym^®^ ELISA with zero incidence of any discrepancy in this population. In those with low clinical suspicion for acute TTP, the AcuStar^®^ misdiagnosed severe ADAMTS13 deficiency (<10 IU/dL), where the parallel ELISA excluded a diagnosis of acute TTP. This discrepancy between the AcuStar^®^ and ELISA assay results was seen in approximately 2% of cases across the participating centres. Notably, there were no reports of false-positive results where a diagnosis of TTP was made based on an AcuStar^®^ result of <1 IU/dL. Although the discrepancy was only seen in a small proportion of cases, the implications for management in such a subgroup are significant in the first instance because it can lead to inappropriate, out-of-region emergency transfers for intensive treatment to a specialist centre for TTP. Further study into the impact of these findings in monitoring patients diagnosed with TTP would provide valuable insight.

Discrepancies between ADAMTS13 activity assays have been reported, commonly when comparing AcuStar^®^ with the FRETS-vWF73 assay. Beranger et al. carried out a large study over two successive sequences: a retrospective evaluation followed by a “real-life” prospective assessment evaluating > 500 citrated plasma samples with a specific focus on levels < 25 IU/dL and overall method comparison [18]. In contrast to the discrepancy trend for false-positive results observed in our report, this study demonstrated the sensitivity of the AcuStar^®^ ADAMTS13 activity assay for acute TTP to be 90.1%, with 14/142 (9.8%) patients with undetectable ADAMTS13 activity misdiagnosed. However, the specificity was >99% [18].

There have been fewer studies evaluating the AcuStar^®^ ADAMTS13 assay against the Technozym^®^ ELISA. Favresse et al. retrospectively reported on 38 citrated plasmas samples, including eight patients with TTP, observing no misclassification (<10% ADAMTS13 activity cut-off) [20]. Valsecchi et al. performed a retrospective and prospective comparison of the AcuStar^®^ ADAMTS13 activity assay with both Technozym^®^ ADAMTS13 activity ELISA and in-house FRETS-vWF73 on a total of 176 citrated plasma samples (including 110 TTP samples) [21]. This study only identified two discrepancies in assessing undetectable (<10 IU/dL) ADAMTS13 activity when compared with the in-house FRETS-vWF73. Stratmann et al. compared the AcuStar^®^ ADAMTS13 activity assay and Technozym^®^ ADAMTS13 activity ELISA on 24 paired citrated plasma samples; these were derived from ten consecutively recruited patients (n = 8, acquired TTP; n = 1, atypical haemolytic uremic syndrome; n = 1, normal control), of which nine test samples were collected from acute TTP patients and 13 samples were collected from TTP patients in clinical remission [19]. Results from this study showed a trend for underestimation by the AcuStar^®^ in the intermediate-low activity range, supporting the data from Favresse et al. [20].

Finally, the most recent study by Singh et al. described 42 samples tested across all available ADAMTS13 testing platforms (FRETS-vWF73, AcuStar^®^, Technozym^®^ ELISA and Ceveron^®^ FRET) [17]. These were from patients with TTP at various time points in the disease course, including acute presentation of other TMAs, and four from critically unwell patients with COVID-19 in the intensive care unit. The group reported median results on the AcuStar^®^ lower than that for the other methods, which had comparable levels. Specifically, the authors highlighted AcuStar^®^ values < 10 IU/dL for five samples from patients who were confirmed to have a non-TTP diagnosis by FRETS; three of five of these false-positive results in the presentation diagnostic samples were in patients who had severe sepsis [17]. In the retrospective analysis of 241 samples with known ADAMTS13 levels demonstrating a high sensitivity for ADAMTS13 levels < 10 IU/dL on the AcuStar^®^, Pascual et al. also found that at least of one of their false positives had sepsis [15]. 

### 4.2. Comparing ELISA and AcuStar^®^ ADAMTS13 Activity Results in the Context of Clinical Index of Suspicion for the Diagnosis of Acute TTP

The PLASMIC and French clinical scoring systems were inconsistently used in the triage of suspected TTP referrals, reflecting the UK’s real-world clinical practice. However, when retrospectively applied, higher scores supported a diagnosis of TTP (Table 1). The presence of a concurrent cancer diagnosis or sepsis was observed in a significant proportion of cases (40%) where a discrepancy in ADAMTS13 results was found (Table 2 and Figure 1). In these cases, the result by AcuStar^®^ was consistently < 10 IU/dL (suggestive of TTP) with an ELISA result > 20 IU/dL (ruling out acute TTP). Furthermore, this group of patients appeared to demonstrate a trend towards more severe thrombocytopenia and had an associated coagulopathy (Table 1). These results highlight the importance of the pre-test clinical probability of an acute TTP diagnosis when interpreting ADAMTS13 activity levels using the AcuStar^®^ assay in the acute setting. Caution should be exercised when interpreting ADAMTS13 AcuStar^®^ results where patients referred acutely are as likely to be unwell due to a concurrent diagnosis of malignancy or an inflammatory condition.

Most patient samples in previous method comparisons have been from pre-selected patients with a high clinical suspicion of TTP and rarely from patients who are presenting with de novo clinical manifestations and plausible alternative diagnoses. Whilst highly dependable in a pre-selected cohort of patients, acute phase reactants may confound the assay in patients with other comorbidities predisposing to microangiopathy of other aetiologies. Excluding acutely unwell patient samples for verification excludes the investigation and identification of interfering substances, biochemical or pharmacological, that may be seen in clinical samples. Interfering substances in vitro are not uncommon in specialist haemostasis testing; for example, the lupus anticoagulant and a reduced one-stage factor assay activity being determined for single-point assays [22]. Possible causes for the discrepancy reported in this cohort of patients are the subject of ongoing investigation [17]. 

### 4.3. Practical Considerations When Utilising the AcuStar^®^ ADAMTS13 Activity Assay in the Diagnosis of Acute TTP

Current guidelines for diagnosing and managing acute TTP lean heavily towards clinical acumen and a constellation of readily available laboratory markers, pending confirmation of an ADAMTS13 activity of <10 IU/dL. ISTH guidelines for the diagnosis of acute TTP stratify ADAMTS13 based on testing availability, with a cut-off for activity measurement of 72 h from the presentation [2]. Rapid assays for ADAMTS13 quantification disrupt this status quo, opening new windows for earlier confirmatory diagnosis and influencing therapeutic decision-making to initiate novel therapeutics such as caplacizumab [23]. 

**Figure 2:**
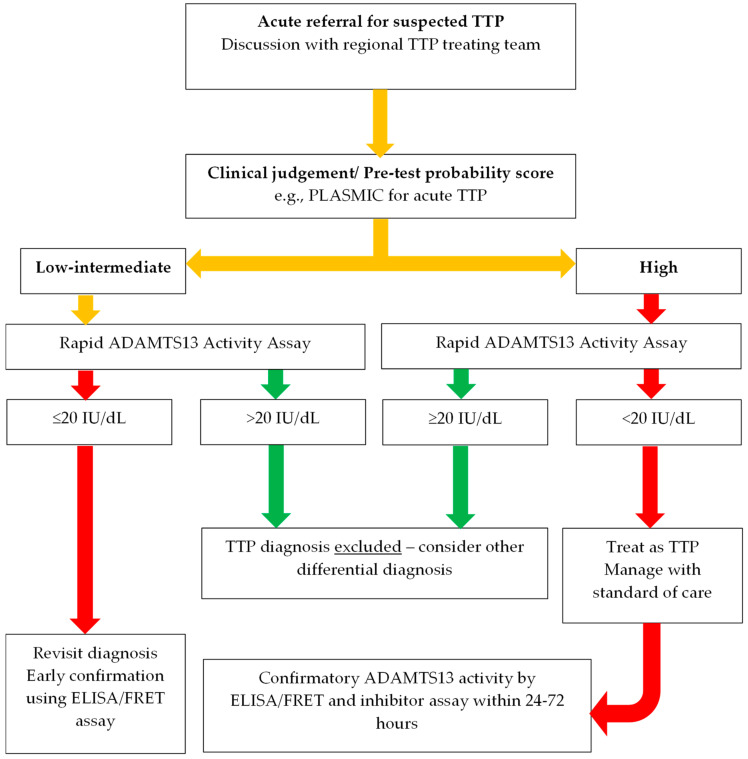
Suggested incorporation of ADAMTS 13 laboratory testing in diagnostic pathways for suspected acute TTP. TTP, thrombotic thrombocytopenic purpura; ELISA, enzyme-linked immunosorbent assay; FRET, fluorescent resonance energy transfer.

Specialist TTP management is increasingly delivered in regional centres where teams familiar with diagnosing this rare condition are perhaps best placed to question any laboratory inconsistencies which may arise. Integration of the rapid assay into non-specialist laboratories may be premature while we are still evaluating the role of a rapid assay within a robust diagnostic framework. Nevertheless, for acutely referred cases, an active dialogue between clinical and laboratory personnel alongside a simple algorithm is paramount in ensuring a consistent diagnostic approach (Figure 2). In the absence of large-scale multi-centre prospective studies, the evidence presented here suggests that in patients where there is low clinical suspicion for TTP, confirmatory testing within 24–72 h with a conventional assay (ELISA/FRETS) should remain in place. 

Limitations of the data presented include their retrospective nature, case note dependence, and the specific comparison of only Technozym^®^ and AcuStar^®^ methods. The evaluation was restricted to testing for acute TTP diagnosis, where rapid ADAMTS13 testing potentially carries the greatest impact. ADAMTS13 monitoring post-acute diagnosis or during stable outpatient follow-up is not subject to the same time pressure. However, an unexpected result in this context should prompt a confirmatory test using a conventional assay. Different assay performances and specifications between established and rapid tests do not have sufficient evidence to assure the transposability of results independent of the analytical platform. Therefore, until more data become available, routine monitoring of patients should ideally occur on a single platform or undergo confirmation using an alternative assay.

## 5. Conclusions

In cases where the clinical presentation supports the diagnosis of TTP, the AcuStar^®^ assay appears to have a valuable role to play in reducing the time to transfer and specialist treatment for acute TTP. In such cases, there is a good correlation between the rapid AcuStar^®^ ADAMTS13 assay and the conventional ELISA assay. Caution should be exercised in the interpretation of the ADAMTS13 activity using the AcuStar^®^ assay where the clinical suspicion for acute TTP is low as the AcuStar^®^ may indicate a false-positive ADAMTS13 deficiency of <10 IU/dL. To avoid misinterpretation of this discrepancy and mismanagement, robust pathways incorporating testing algorithms should be available in TTP treating centres and laboratories performing these tests. 

## Figures and Tables

**Table 1:** Characteristics of discrepant ADAMTS13 vs. non-discrepant groups (ELISA vs. AcuStar^®^ rapid assay).

	Discrepant ADAMTS 13 (N = 20) (Median, Q1–Q3)	Non-Discrepant ADAMTS13 (N = 20) (Median, Q1–Q3)	*p* Value
Demographics			
Age (years)	58 (51.5–75)	42 (28–54)	0.009
Female sex (%)	55	85	0.088
Clinical parameters			
PLASMIC score	4 (4–4)	6 (5–7)	<0.001
French TMA score	1 (0–1)	2 (2–2)	0.004
Cancer/transplant comorbidity (%)	15	5	0.302
Admission laboratory parameters			
Platelet count (×10^9^/L)	55 (27–89)	13 (10–21)	0.199
LDH (U/L)	1187 (943–1506)	854 (451–1326)	0.108
Creatinine (mmol/L)	205 (175–238)	72 (65–87)	0.003
INR	2.30 (1.50–3.35)	1.09 (1.07–1.11)	<0.001
Fibrinogen (g/L)	3.05 (1.33–4.85)	3.07 (2.40–3.96)	0.358
Diagnostic results			
AcuStar^®^ CIA ADAMTS13 activity (IU/mL), MRR 60–130.6% *	8.3 (6.4–11.3)	4.2 (2.7–8.1)	<0.001
ADAMTS13 activity by ELISA (IU/mL), MRR 40–130 IU/dL **	42 (29–50)	5 (2–7)	<0.001
ADAMTS13 inhibitor, MRR > 11 U/mL	5.1 (2.8–6.1)	44.5 (17.5–75.5)	<0.001

This table uses manufacturer’s reference range for reference. Local reference ranges were employed across centres. Q, quartile; TMA, thrombotic microangiopathy; LDH, lactate dehydrogenase; INR, international normalised ratio; PT, prothrombin time; aPTT, activated partial thromboplastin time; CIA, chemiluminescent immunoassay; MRR, manufacturer reference range. * Manufacturer reference range quoted as %. ** Manufacturer reports reference range as IU/mL; conversion to IU/dL has been made for the purposes of comparison. Results are presented as median (IQR) unless otherwise stated for proportions or counts.

**Table 2:** Comorbidities of patients upon retrospective analysis of records, discrepant ADAMTS13 vs. non-discrepant ADAMTS13 results.

Comorbidity	Number of Patients with Comorbidity (n)	
	Discrepant ADAMTS13	Non-Discrepant ADAMTS13
Sepsis	5	0
COVID-19 infection	3	0
Acute kidney injury	4	0
Ischaemic stroke	3	0
Cancer, active	3	0
Cancer, remission	0	1
Previous stem cell transplant	0	1
DIC	2	0
Ischaemic heart disease	1	0
HELLP syndrome	1	0
Previous TTP	0	4

Those with non-discrepant ADAMTS13 results had a high clinical suspicion of TTP. Patients may have more than a single comorbidity listed. COVID-19, coronavirus disease 2019; DIC, disseminated intravascular coagulation; HELLP, haemolysis, elevated liver enzymes, and low platelets; TTP, thrombotic thrombocytopenic purpura.

## Data Availability

Data is available upon request to the corresponding author.
